# Modeling and Simulation of the Transient Response of Temperature and Relative Humidity Sensors with and without Protective Housing

**DOI:** 10.1371/journal.pone.0095874

**Published:** 2014-05-22

**Authors:** Keller Sullivan Oliveira Rocha, José Helvecio Martins, Marcio Arêdes Martins, Ilda de Fátima Ferreira Tinôco, Jairo Alexander Osorio Saraz, Adílio Flauzino Lacerda Filho, Luiz Henrique Martins Fernandes

**Affiliations:** 1 Departamento de Engenharia Agrícola, Universidade Federal de Viçosa, Viçosa, Brazil; 2 Departamento de Ingeniería de Alimentos, Universidad Nacional de Colombia, Medellín, Colombia; University of Milano-Bicocca, Italy

## Abstract

Based on the necessity for enclosure protection of temperature and relative humidity sensors installed in a hostile environment, a wind tunnel was used to quantify the time that the sensors take to reach equilibrium in the environmental conditions to which they are exposed. Two treatments were used: (1) sensors with polyvinyl chloride (PVC) enclosure protection, and (2) sensors with no enclosure protection. The primary objective of this study was to develop and validate a 3-D computational fluid dynamics (CFD) model for analyzing the temperature and relative humidity distribution in a wind tunnel using sensors with PVC enclosure protection and sensors with no enclosure protection. A CFD simulation model was developed to describe the temperature distribution and the physics of mass transfer related to the airflow relative humidity. The first results demonstrate the applicability of the simulation. For verification, a sensor device was successfully assembled and tested in an environment that was optimized to ensure fast change conditions. The quantification setup presented in this paper is thus considered to be adequate for testing different materials and morphologies for enclosure protection. The results show that the boundary layer flow regime has a significant impact on the heat flux distribution. The results indicate that the CFD technique is a powerful tool which provides a detailed description of the flow and temperature fields as well as the time that the relative humidity takes to reach equilibrium with the environment in which the sensors are inserted.

## Introduction

Technological development with respect to instrumentation systems and measures increasingly demands the means to provide greater reliability of the data collected.

Computational fluid dynamics (CFD) simulations are increasingly used to predict various issues in agriculture and food processing [1]–[5]. The performance of agribusiness or agricultural processes depends on the heat exchange between air and water and the products or animals, and, therefore, on the spatial distribution of air parameters, such as speed, turbulence, temperature and relative humidity [6]–[8].

Protective housing for temperature and relative humidity sensors is normally required to prevent damage due to the physical and environmental influences on agricultural processes. A major concern about protected temperature and relative humidity sensors is the response time delay in a process reaching equilibrium with its environment, which may affect its accuracy if the measurement is taken at an inadequate time.

The main objective of this work was to identify how much time it takes for temperature and relative humidity sensors to respond properly in a dynamic varying process, under certain specified operational conditions.

## Methodology

As a first step, experiments were performed in a wind tunnel, and CFD simulations, to predict the distributions of temperature and relative humidity in a non-isothermal flow, were accomplished.

**Figure 1:**
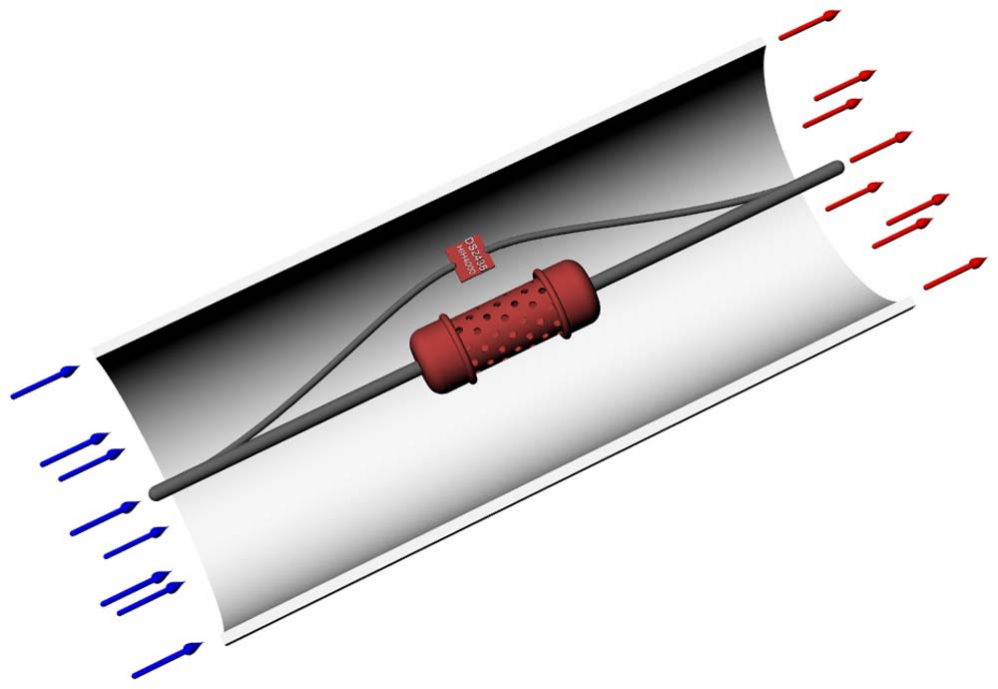
Schematic representation of the wind tunnel with temperature and relative humidity sensors.

A small wind tunnel, 200 mm in diameter and 500 mm high, was designed to provide a laminar flow with a speed between 0.5 and 1 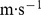, as shown in Figure 1.

The investigation of the heat exchange and mass transfer between the environment and the housing was divided into two parts: (1) a study of the interface between the fluid medium (air) outside and inside the housing, (2) analysis of the fluid flow (air) at constant speed for high temperature (77.5°C), external to the housing.

Measurements were taken at the point closest to the center outside the housing and at the center point inside. The Reynolds number 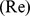 at these points was, approximately, 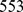 and 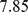, respectively, and can be described by Navier–Stokes equations.

Results were obtained from the standard 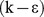 turbulence model, and the model equation for heat transfer was compared to the wind tunnel experiment.

In order to gain further insight into the heat transfer and mass structures of PVC (polyvinyl chloride) materials and to analyze the behavior of the temperature and relative humidity sensors more deeply, CFD model simulations were performed to investigate the distribution of temperature and relative humidity in the transient regime at the observation points, and the findings were compared with results obtained in the wind tunnel.

### Study of the interface between the fluid medium outside and inside the housing

#### Operating conditions

A data acquisition system, based on 1-Wire technology, with real-time monitoring was implemented using addressable digital devices [9], [10]. The use of such a network for data acquisition is interesting, because it reduces the cost of instrumentation, since it is not necessary to use conventional data acquisition systems.

The 1-Wire network uses only a conductor for data transmission, in common with all instruments or devices (slaves) connected to the network, and a computer or microprocessor (master), which manages the whole system through an appropriate computer program, and is technically and economically feasible [11]–[14].

The temperature and relative humidity in the wind tunnel were measured at two central points. A sensor without protective housing was placed close to the wind tunnel center and exposed to the air stream. Another sensor with protective housing was installed at the center of the wind tunnel and received the airflow through the perforations made in the housing wall. The average airflow rate was held constant through the wind tunnel. External environmental variables, such as temperature and relative humidity, were also monitored with a sensor without housing.

**Figure 2:**
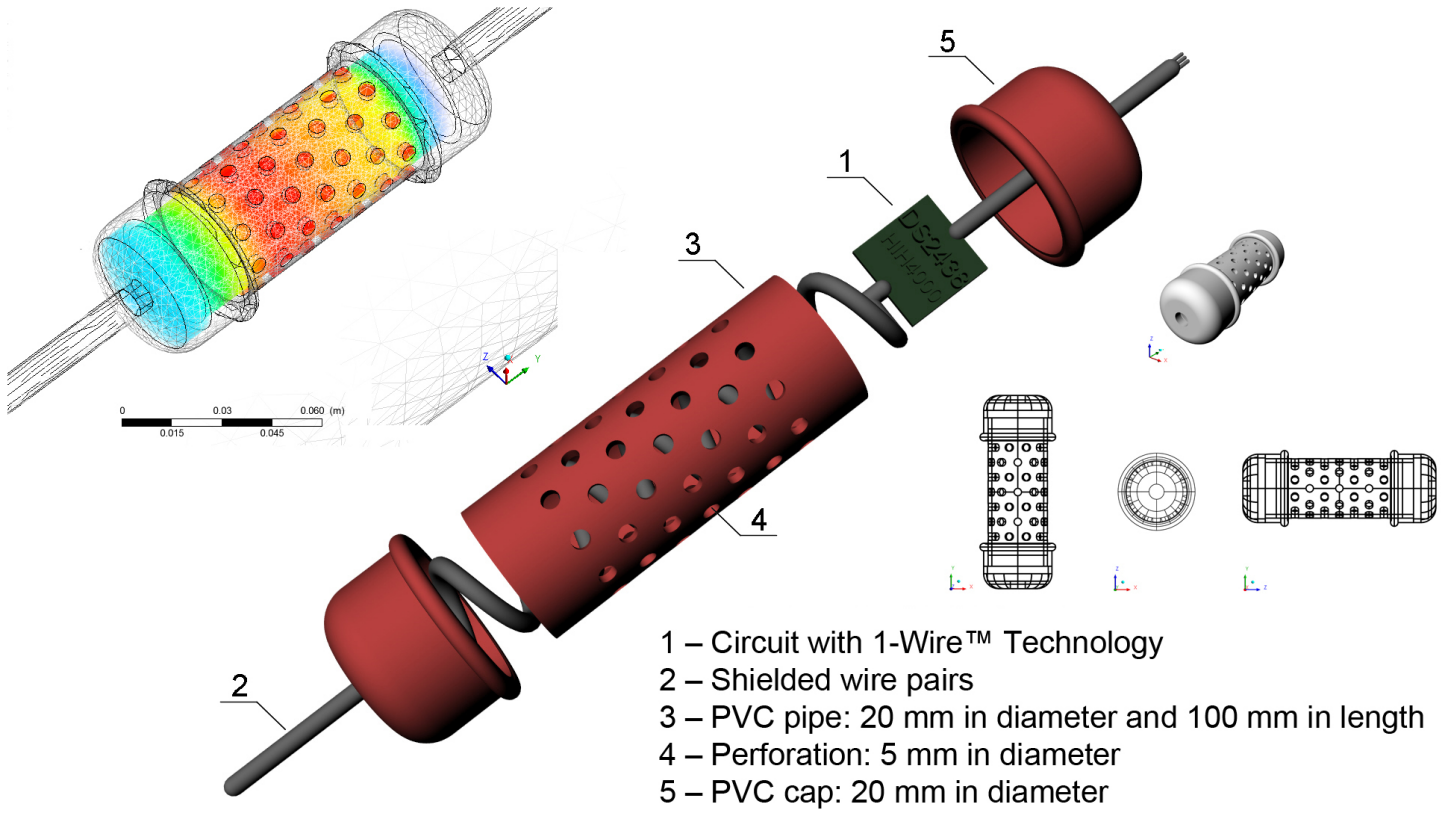
Schematic representation of the housing for the temperature and relative humidity sensors.

The housing for the circuit with a temperature sensor (DS2438) and a humidity sensor (HIH4000) with 1-Wire technology inside the wind tunnel was constructed using a PVC tube 100 mm long with a diameter of 32 mm. Perforations of 5 mm diameter were drilled into the housing wall to allow for airflow around the sensors, connected by shielded wire pairs, as illustrated in Figure 2.

#### Experimental data acquisition

A data acquisition system was programmed using the 1-Wire protocol and based on a system called STRADA [13].

As previously discussed, one sensor with protective housing and one without were installed inside the wind tunnel. A third sensor was installed outside the tunnel to monitor the external environment conditions. The sensors were interconnected by a four-way telephone wire to acquire and transmit data [11].

The temperature and relative humidity data were recorded with a resolution of 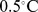 and 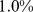, respectively.

The airflow speed in the wind tunnel was set at 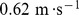 at a maximum temperature limit of 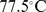. At the beginning of the heating process, the average of both temperature and relative humidity in the external environment were 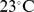 and 65%, respectively.

### Computational model

Due to the complexity of the housing geometry, the ANSYS ICEM CFD software was used to construct a tetrahedral computational mesh of the object under study.

The tetrahedral volume element is very useful for maintaining the high quality of the mesh while articulating different forms of complex geometry to be modeled in the region of interest, even though it requires more nodes than the mesh elements and results in a hexahedral file size and increased computation time [15], [16].

### Boundary conditions

The results obtained by simulation using CFD were verified and compared with the corresponding data obtained experimentally in the wind tunnel.

The measured values and those obtained experimentally in the wind tunnel were used to assign the boundary conditions of the model. Thus, it was necessary to provide the initial values of variables such as relative humidity and air temperature inside and outside the enclosure, calculate the humidity and temperature for each step in time, and solve the equations of conservation of energy, mass and momentum.

The CFD technique was used to solve the Navier–Stokes equations written for the velocity and energy fields, quantifying the fields of temperature and velocity by the finite volume method [4], [17]–[20]. These equations are presented below.
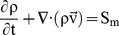
(1)

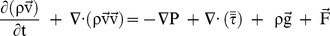
(2)

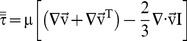
(3)

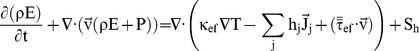
(4)

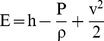
(5)

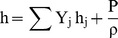
(6)

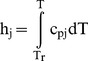
(7)where:

h - Convective heat transfer coefficient 
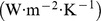


h_m -_ Mass transfer coefficient 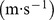


P – Pressure (Pa)

T – Temperature (K)

T – Time (s)


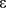
_ -_ Dissipation rate of speed fluctuations 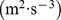


k - Turbulent kinetic energy coefficient 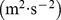



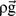
 - Gravitational force of the body per unit volume 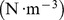



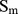
- Mass added to the continuous phase 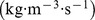



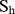
- Mass added or removed from the continuous phase 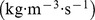



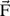
- External forces of the body per unit volume 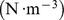



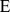
- Specific energy 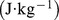



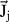
- Diffusion flux of species j 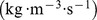



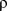
- Density 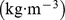



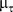
- Dynamic viscosity of the fluid 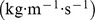



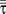
- Stress tensor 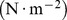



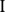
 - Tensor unit


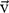
- Flow velocity vector 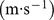



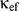
- Effective thermal conductivity 
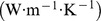


The Reynolds tensor was modeled using the standard turbulence 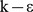 model, evaluating the viscosity (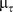) from the relationship between the kinetic energy of turbulence (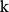) and dissipating kinetic energy of turbulence (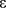).

After exposure of the sensors to the airflow for a specific period of time for a given level of humidity, a relative humidity value is reached, which depends on the temperature and the amount of water contained in the air at the equilibrium state.

Relative humidity is a measure of the amount of water contained in the air, defined by the ratio between the actual pressure of water vapor in the atmosphere and the pressure of saturated water vapor in the atmosphere at the same temperature, usually expressed as a percentage.
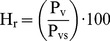
(8)where:


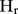
- Relative humidity (decimal or %)


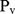
- Pressure of water vapor in the atmosphere (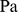)


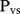
- Pressure of saturated water vapor in the atmosphere (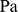)

The empirical relationship between the saturation vapor pressure and the temperature is given by the following equation [21]:
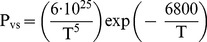
(9)


The ANSYS CFX computer program was used to implement the proposed model with the following assumptions:

1. Transportation in transient state.
2. Incompressible flow.

A residue with a value less than 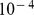 was adopted as the convergence criterion.

### Validation of the computational model

The variables were tested for normality using the Shapiro–Wilk test and subsequently an analysis of variance was carried out. Comparisons between independent replications were performed by analysis of variance for data with a normal distribution and by the nonparametric Kruskal–Wallis test for data without a normal distribution.

After the appropriate transformations, data were submitted for nonparametric testing using the SigmaPlot, version 11.0, computer program, from Systat Software Inc. [22], for statistical analysis and data representation. The probability level (p-value) was fixed at 5% in order to obtain a reliability of 95% in the comparisons.

## Results and Discussion

This work was developed at the Departamento de Engenharia Agrícola, Universidade Federal de Viçosa, Viçosa, State of Minas Gerais, Brazil.

**Figure 3:**
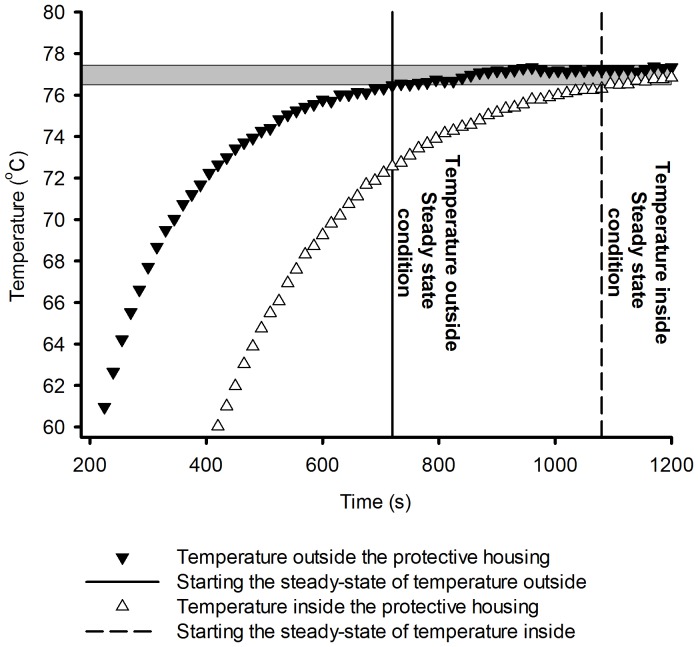
Difference between the time that the airflow temperature outside and inside the sensor's housing takes to reach the equilibrium.

**Figure 4:**
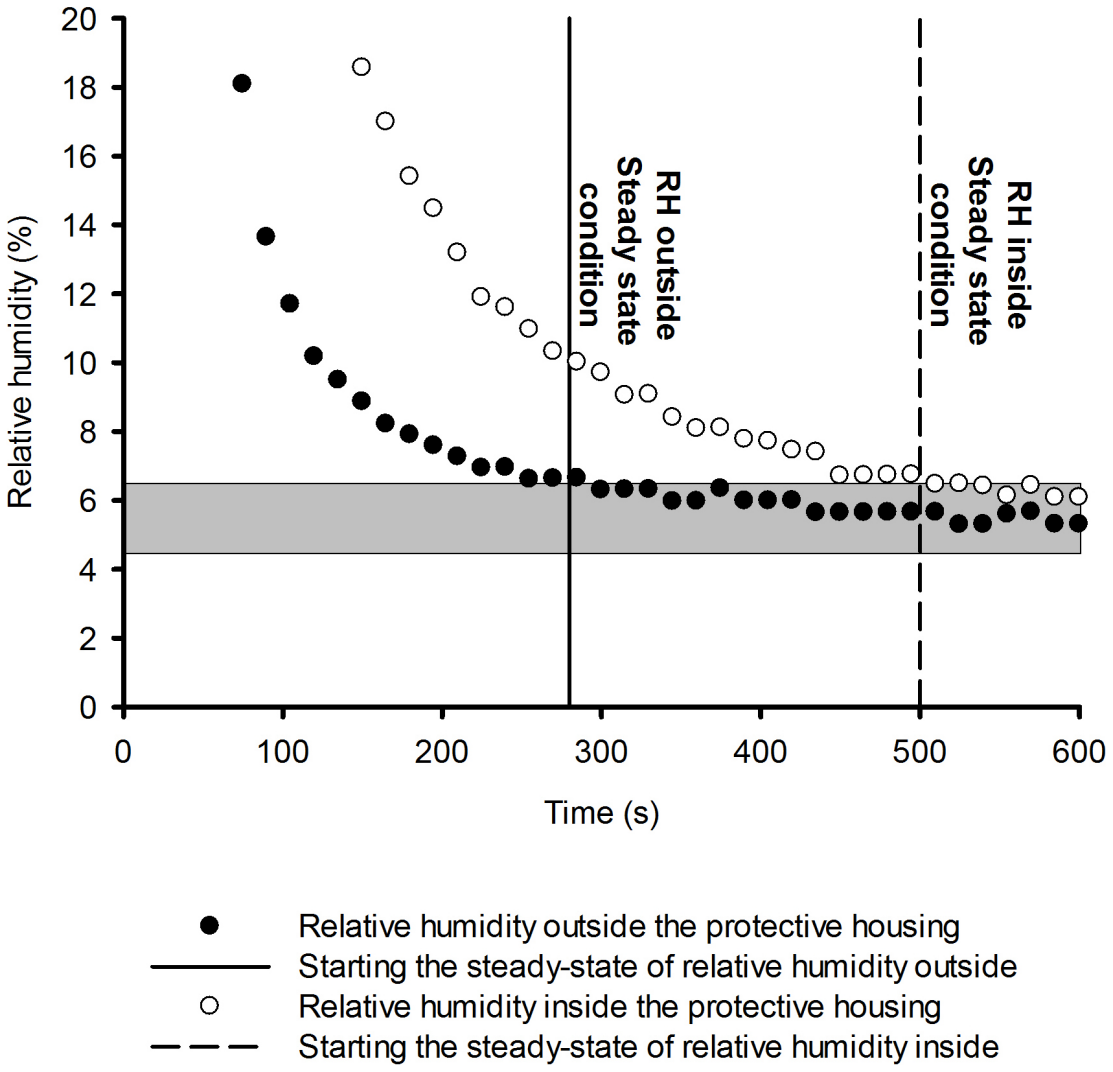
Difference between the time that the air relative humidity outside and inside the sensor's housing takes to reach the equilibrium.

To solve the problem of heat and mass transfer using CFD, first the behavior of the fluid (air) under two experimental conditions (external and internal to the protection housing) was analyzed. The behavior of the temperature variation is shown in Figure 3 and the relative humidity in Figure 4.

After the experimental behavior of the fluid (air) in the proposed scenarios was analyzed, the process was modeled. The boundary conditions took into account the temperature at the entrance of the control volume.

### Modeling of the process

The temperature of the fluid stream outside the protective sensor's housing was measured, starting at instant zero corresponding to the ambient air temperature until a maximum steady temperature was reached, according to the power and airflow rate of the fan used. A mathematical model was fitted to the experimental data by nonlinear regression analysis. The model that best fitted to the experimental data was an exponential type equation, expressed by Equation 10, which is similar to the models found in the literature for this kind of process [23].
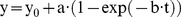
(10)where:

y - Temperature (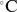)


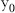
 - Temperature at time zero (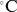)

t - Time (s)

a, b - Regression coefficients

The coefficient estimates of the fitted model, along with their standard error, t-statistics values, and probability significance level, are presented in Table 1.

**Table 1:** Coefficient estimates and statistical analysis of the fitted model.

Parameter	Estimate	Standard error	t-value	p-value
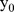	25.3864	0.3799	66.8183	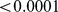
a	51.507	0.3746	137.4827	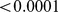
b	0.0083	0.0001	79.6398	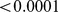

The adjusted coefficient of determination (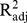) and the standard error of the estimates (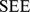) were 0.9971 and 0.5976, respectively.

The analysis of variance for the regression model is presented in Table 2.

**Table 2:** Analysis of variance for the regression model.

Factor	DF	SS	MS	F	p-value
Regression	2	9678.7848	4839.3924	13552.4554	<0.0001
Residual	79	28.2098	0.3571		
Total	81	9706.9946	4839,7495		

The sample data set used in the regression analysis was tested for normality using the Shapiro–Wilk test and passed with p = 0.0388 and W = 0.9681 with a significance level ≤0.0001.

**Figure 5:**
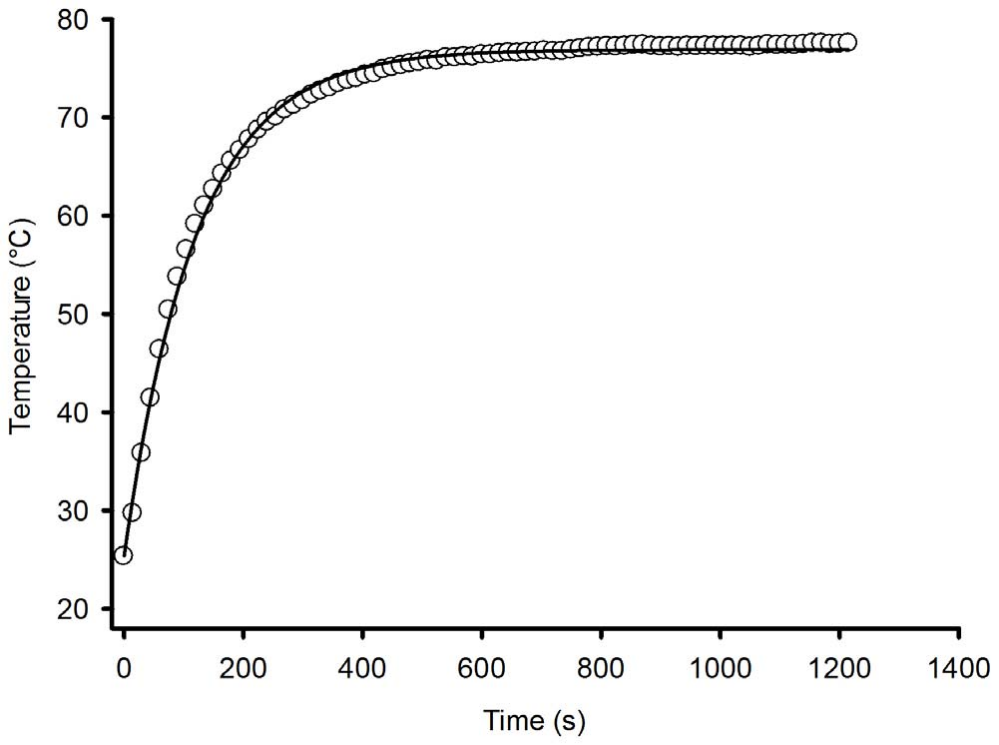
Variation of measured and estimated airflow temperatures outside the protective sensor's housing during the heating process.

**Figure 6:**
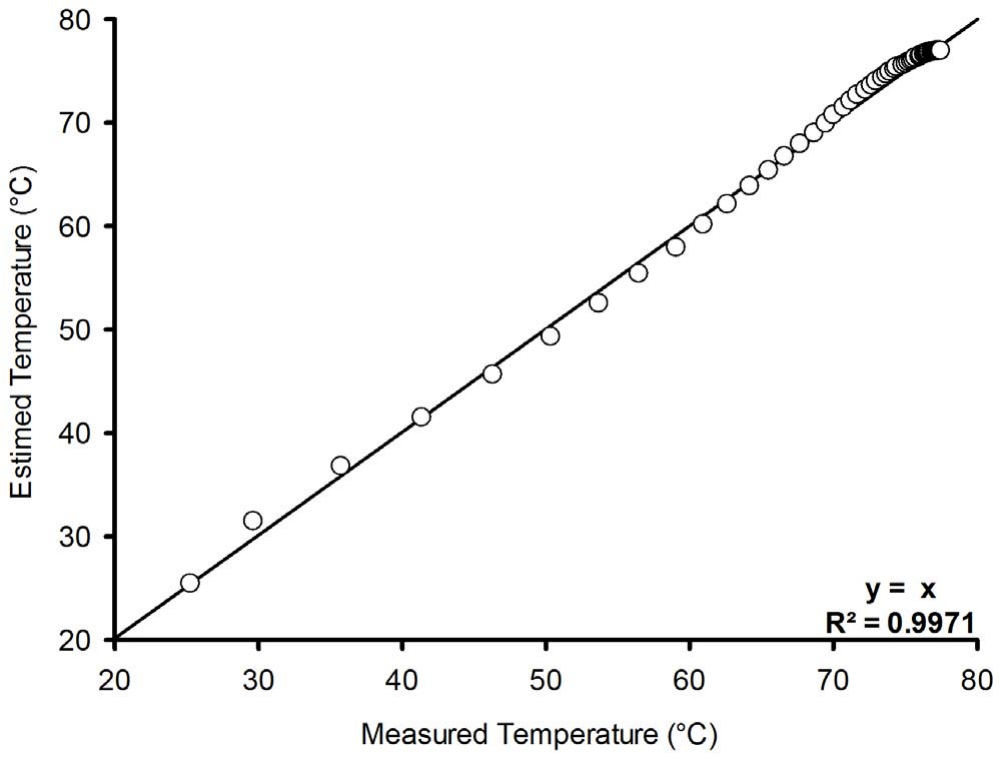
Comparison between estimated and measured airflow temperatures outside the protective sensor's housing during the heating process.

Analyzing all factors presented in Tables 1 and 2, and the adjusted coefficient of determination 
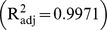
, it can be concluded that the fitted model estimates the variation of the air temperature outside the protective sensor's housing very well, as can be observed in Figures 5 and 6.

Therefore, based on the previous analysis, the fitted model can be safely used to estimate the input conditions (boundary conditions) for predicting the response of temperature and relative humidity sensors inside the protective housing. This is very important to determine the exact instant at which the measurements should be taken to obtain the correct, or at least, a satisfactory value of the measure of interest, principally if the process is taking place in a hostile or dirty environment.

### Simulation study

The model previously discussed was used to estimate the boundary conditions (input conditions) of the simulation model.

**Figure 7:**
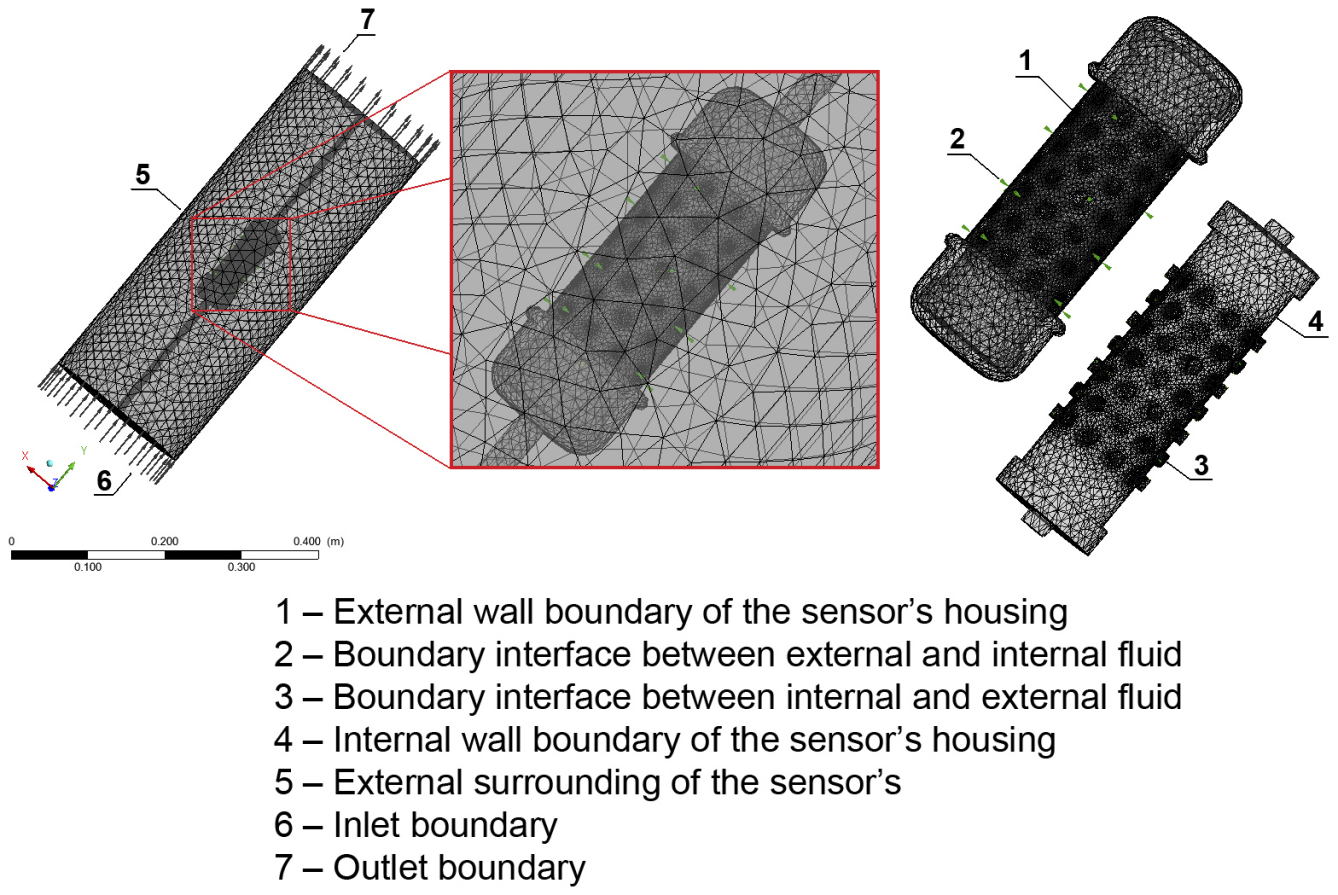
Detail of computational tetrahedral mesh refinement.

In order to accomplish the simulation, ICEM ANSYSTM software was used to generate meshes for the system. A tetrahedral mesh was created with a total of 356,654 finite volumes comprising two distinct domains: (1) formed by the fluid confined in the protective housing consisting of 217,069 finite volumes, and (2) formed by the unconfined fluid consisting of 139,585 finite volumes. Both domains were interconnected by a fluid-type interface at the openings in the PVC tube. Boundary conditions were established for each face of each domain, as shown in Figure 7.

Using Equation 10 to furnish the input boundary conditions, the CFD technique was used to solve the Navier–Stokes equations written for the velocity and energy fields (Equations 1 to 7), quantifying the fields of temperature and velocity by the finite volume method [4], [17]–[20].

**Figure 8:**
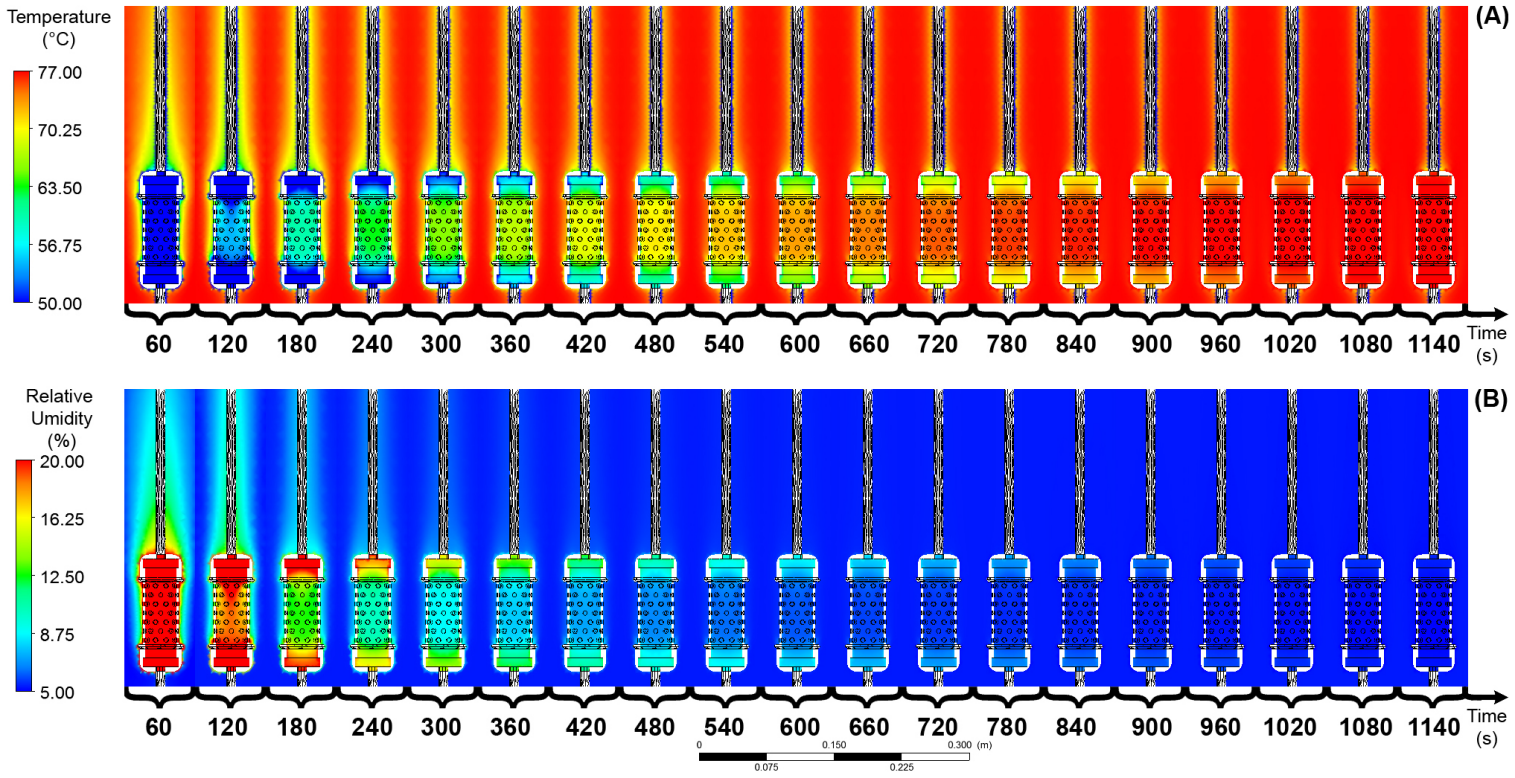
(A) Temperature variation, and (B) relative humidity variation. (Video S1).

During the study period (1,140 s), it was observed that the time for temperature and relative humidity to reach the equilibrium conditions for sensors with and without protective housing differed by 1,020 s and 540 s, respectively, with a variation of 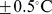 and 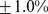. An illustration of this process is shown in Figure 8 (A and B).

**Figure 9:**
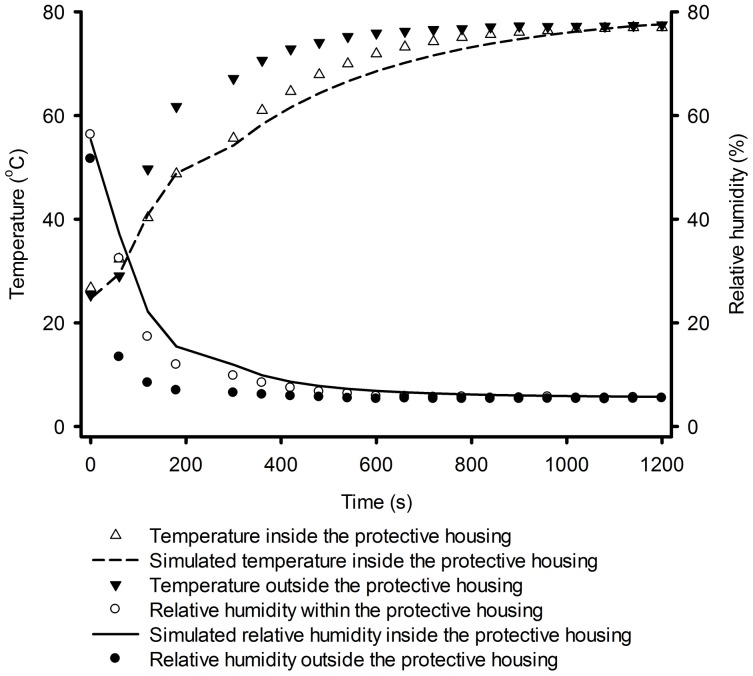
Simulation of the transient state of temperature and relative humidity for sensors with protective housing.

Figure 9 shows the temperature and relative humidity, both measured outside the sensor's housing (black triangle and dots), and also the measured and predicted results inside the housing (white triangles and dots, and dashed and continuous line).

Figure 9 illustrates clearly the difference between the time that the airflow temperature outside and inside the sensor's housing takes to reach the pre-established value. These times were, approximately, 720 s and 1100 s, respectively. It also illustrates the same for the air relative humidity. In this case, the times were, respectively, 280 s and 500 s.

It should be pointed out that these results are for a particular case and, certainly, may vary for other conditions or processes. However, they give an indication of how to proceed when measuring temperature and relative humidity, for example, in the mass of a biological product, such as cereal grains.

**Figure 10:**
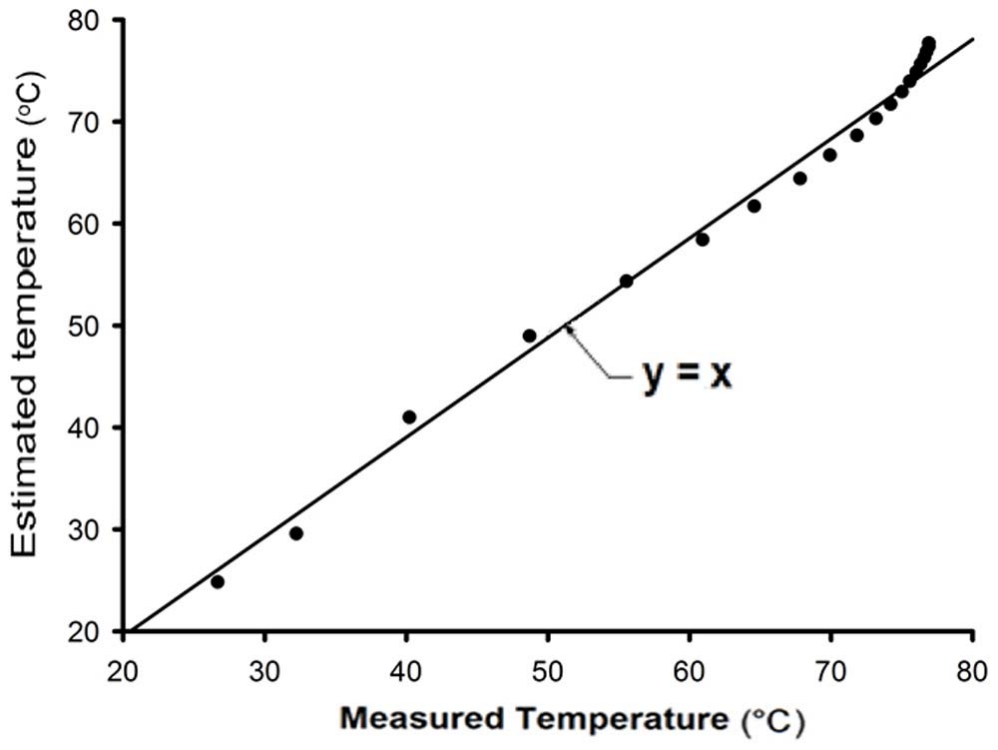
Comparison between measured and simulated temperatures inside the sensor's housing.

**Figure 11:**
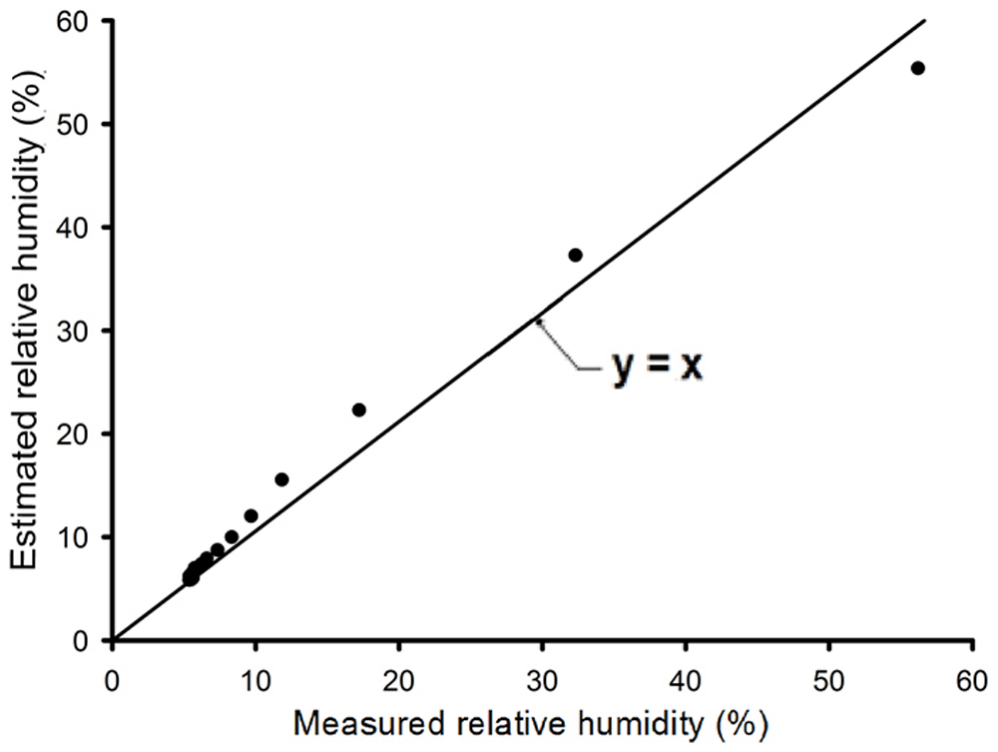
Comparison between measured and simulated relative humidity inside the sensor's housing.

A high correlation was found between the measured and simulated temperature inside the sensor's housing, as well as between the measured and simulated relative humidity inside the sensor's housing, as shown in Figures 10 and 11, respectively.

It can be seen in Figure 10 that there is a discrepancy between the estimated and the measured values for temperatures above 75°C. This is due to the decrease in the sensor's accuracy above this temperature. Therefore, to measure temperature above this value, another type of sensor should be used. However, for the majority of agricultural and engineering processes, this range of temperature is appropriate. For relative humidity, as shown in Figure 11, this discrepancy was not observed.

## Conclusions

As a material of high resistance and low cost, PVC tubes are appropriate for housing sensors exposed to hostile environments.

The results obtained from simulations of temperature and relative humidity show a good correlation with experimental data (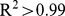), which indicates that the model is appropriate for use in improving housing sensor systems using PVC material.

The mean time to reach the equilibrium temperature and relative humidity sensors with and without housing is 1,020 and 540 s, respectively, with a variation of ±0.5°C, and 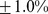.

This methodology can be used as a basis for the initial design of new morphologies and geometries in order to optimize the flow inside the enclosure and reduce the response time (equilibrium).

## Supporting Information

Video S1 **Temperature and relative humidity variation.**
(AVI)
