# Correction: Modeling and Simulation of the Transient Response of Temperature and Relative Humidity Sensors with and without Protective Housing

**DOI:** 10.1371/journal.pone.0100485

**Published:** 2014-06-12

**Authors:** The PLOS ONE Staff

There is an error in the funding statement of this article that was introduced during the preparation of this manuscript for publication. The correct Funding statement is:

“The work was funded by CAPES - Coordenação de Aperfeiçoamento de Pessoal de Nível Superior/PROEX - Programa de Excelência Acadêmica (AUX CAPES/PROEX 1534/2013 -http://www.capes.gov.br/bolsas/bolsas-no-pais/proex), CNPq - Conselho Nacional de Desenvolvimento Científico e Tecnológico and FAPEMIG - Fundação de Amparo à Pesquisa do Estado de Minas Gerais. The funders had no role in study design, data collection and analysis, decision to publish, or preparation of the manuscript.”
